# Development and Evaluation of Sustained Release Tablet of Betahistine Hydrochloride Using Ion Exchange Resin Tulsion T344

**DOI:** 10.5402/2012/438342

**Published:** 2012-06-18

**Authors:** Vijay D. Wagh, Nilesh Pawar

**Affiliations:** Department of Pharmaceutics, R. C. Patel Institute of Pharmaceutical Education and Research, Near Karvand Naka, Maharashtra, Shirpur 425405, India

## Abstract

An attempt was made to sustain the release of Betahistine hydrochloride by complexation technique using strong cation-exchange resin, Tulsion T344. The drug loading onto ion-exchange resin was optimized for mixing time, activation, effect of pH, swelling time, ratio of drug : resin, and temperature. The resinate was evaluated for micromeritic properties and characterized using XRPD and IR. For resinate sustained release tablets were formulated using hydoxypropyl methylcellulose K100M. The tablets were evaluated for hardness, thickness, friability, drug content, weight variation, and in vitro drug release. Tablets thus formulated (Batch T-3) provided sustained release of drug over a period of 12 h. The release of Betahistine HCl from resinate controls the diffusion of drug molecules through the polymeric material into aqueous medium. Results showed that Betahistine HCl was formulated into a sustained dosage form as an alternative to the conventional tablet.

## 1. Introduction

Betahistine hydrochloride is an orally administered antihistaminic drug. The chemical name of Betahistine is N-methyl-2-(pyridin-2-yl)-ethanamine. Betahistine has a very strong affinity for histamine H3 receptors and a weak affinity for histamine H1 receptors. It has been used to control vertigo in patients of Meniere's disease; it possibly acts by causing vasodilation in the internal ear. However short biological half-life of Betahistine HCl (2-3 h) necessitates frequent (four times a day) administration of the drug [1–3].

Ion exchange resins have versatile properties as drug delivery vehicles and have been extensively studied in the development of novel drug delivery systems. Cation exchange resins containing strong sulfonic acid group form a strong bond with cationic drugs, and elution of drug from resinate is slower [4]. Ion exchange resins are cross-linked, water insoluble, polymer-carrying, ionizable functional groups. Drugs can be loaded onto the resins by an exchanging reaction, and, hence, a drug-resin complex (drug resinate) is formed [5]. Ion exchange can be defined as a reversible process in which ions of like sign are exchanged between liquid and solid, a highly insoluble body in contact with it [6]. The drug is released from the resinates by exchanging with ions in the gastrointestinal fluid, followed by drug diffusion. Being high molecular weight water insoluble polymers, the resins are not absorbed by the body and are therefore inert [7].

The present research was directed towards the development of sustained release dosage form of Betahistine HCl using ion exchange resin with incorporation of polymer matrix. Tulsion T344 IER and hydroxypropyl methylcellulose (HPMC) K100M were used. Tulsion T344 is a strong cation exchange resin with SO^3−^H^+^ functionality which exchanges cations stoichiometrically in an equilibrium-driven reaction. Due to the presence of SO^3−^H^+^ group resin shows ionization at all body pH values. However simple drug-resin complexes may not satisfy the requirement of sustained release; in such cases resinates are incorporated into the matrix systems, microencapsulated or coated. HPMC is mixed alkyl hydroxypropyl cellulose ether containing methoxyl and hydroxypropyl groups. The hydration rate of HPMC depends on the nature of these substituent's HPMC-tablets hydrates upon contact with water, and a rate-controlling gel layer forms around a solid inner core. A rapid formation of the gel layer is a prerequisite for the retardation of the drug release; otherwise, hydrophilic drugs would be released rapidly [8].

## 2. Materials and Methods

Betahistine Hydrochloride-IP was received as a gift sample from Meridian Medicore Ltd., Baddi India. Tulsion T344 was obtained from Thermax Ltd., Pune, India. MCC (pH 102), and HPMC (K100M) was obtained from Loba Chemie, Mumbai, India. All other chemicals and reagents used were of high analytical grade.

### 2.1. Preparation of Drug-Resin Complex (DRC)

The batch process was used for complexation; DRC was prepared by placing 100 mg of activated resin in a beaker containing 25 mL deionized water. Betahistine (100 mg) was added to resin slurry with magnetic stirring. On filtration, the residue was washed with 75 mL of deionized water. Unbound drug in filtrate was estimated at 260 nm.

### 2.2. Optimization of Concentration of Resin for Drug Loading

The resin with highest amount of drug loading was then optimised for various drug : resin ratio varying from 1 : 1 to 1 : 4. The ratio with maximum drug loading was the optimized ratio. Separate batches of drug resin complex were soaked in 25 mL of deionized water.

### 2.3. Optimization of Stirring Time on Drug Loading

Drug resin complex slurred in 25 mL of deionized water was processed at different stirring time, that is, 30, 60, 120, 180, and 240 minutes. The time required for maximum drug loading was optimized.

### 2.4. Optimization of Swelling Time on Drug Loading

Drug resin complex slurred in 25 mL of deionized water was processed at different swelling time, that is, 15, 30, 45, and 60 minutes. The time required for maximum drug loading was optimized.

### 2.5. Optimization of Temperature on Drug Loading

Drug resin complex slurred in 25 mL of deionized water was processed at different temperatures, that is, 27, 40, 60, and 90°C. The time required for maximum drug loading was optimized.

### 2.6. Effect of pH on Drug Loading

A series of solutions were prepared which contained fixed quantity (400 mg) of resin, in 25 mL of deionised water and about 100 mg of Betahistine. The pH of loading solution was adjusted at 3, 4, 5, 6, 7, and 8 and stirred on magnetic stirrer for 4 hours. DRC was collected by filtration, washed with 75 mL of deionized water to remove uncomplexed drug, and dried at 60°C. Drug content was determined as mentioned previously [9].

### 2.7. Evaluation of Drug Resinate Complex

#### 2.7.1. Micromeritic Properties

 Different physical parameters of resins and DRC like flow properties, bulk density, tap density, and packing ability were studied [10].

#### 2.7.2. FTIR Spectroscopic Studies

 Infrared spectra of DRC, Betahistine HCl, and resin were obtained using Fourier transform infrared (FTIR) spectroscopy (JASCO 460 PLUS, Mumbai) by diffused cell technique. The spectra were recorded over the wave number.

#### 2.7.3. X-Ray Diffraction Studies

Betahistine, resin, and DRC were subjected to X-ray diffraction studies for confirmation of complex formation. The instrument used was Philips Analytical X-ray BV (PW 1710). The samples were irradiated with monochromatized Cu radiations and analyzed between 2*θ* to 60*θ*.

### 2.8. Formulation of Tablets

Composition of different formulations as per Table 1 was prepared using varying amounts of the polymers. The preparation process involved two steps; first DRC was wet granulated with PVP K30 in isopropyl alcohol as a granulating agent. The granules were dried at 60° and pass the granules through 30#. Second step involves blending of granules with HPMC and microcrystalline cellulose. Then granules were lubricated with magnesium stearate. The tablets were compressed into flat-faced punches of 8 mm diameter using Rimek Mini Press-II MT tablet compression machine.

### 2.9. Tablet Assay and Physical Evaluation

Drug content of all the batches was determined. For this purpose ten tablets were weighed and crushed in a small glass mortar with pestle. The fine powder was weighed to get 100 mg equivalent of Betahistine HCl, transferred to 250 mL conical flask containing 100 mL of 1 N HCl, and stirred for 4 h on magnetic stirrer. Dispersion was filtered, and the filtrates obtained were analyzed spectrophotometrically. Tablets were also evaluated for uniformity of weight and thickness. Tablets were examined for friability using a Roche type friabilator and hardness using a Monsanto type hardness tester.

### 2.10. In Vitro Dissolution Studies:

In vitro dissolution study of tablets performed using USP XXIV type II dissolution apparatus (37 ± 0.5, 900 mL, 100 rpm) in phosphate buffer pH 6.8 for a period of 12 h. Aliquots were taken out at every 1 hour for 12 h, and the volume was replaced with an equivalent amount of aliquots of fresh dissolution medium. The samples withdrawn were analyzed. In vitro release of formulation was calculated using PCP Disso software (PCPD V 208). The computed values of kinetic constant (*k*) and diffusional release exponent (*n*) were determined.

## 3. Results and Discussion

Betahistine hydrochloride was loaded on ion exchange resin by batch process. Complexation is essentially a process of diffusion of ions between the resin and surrounding drug solution. As reaction is equilibrium phenomenon, maximum efficacy is best achieved in batch process. Complexation between drug and resin was found to be optimum after 4 h of mixing in all the resins investigated. Highest drug binding on resin was achieved when activated with 1 N HCl and 1 N KOH. The drug loading was found to be 70.32 ± 0.64 for Tulsion T344, respectively. After activation with acid and alkali treatment, the exchangeable ion on the resin is H^+^. Relative selectivity of H^+^ is less than other ionic forms, and therefore it increases percent complexation. Maximum drug loading on the resin occurs at pH 5; a maximum of 94.19 ± 0.78 for Tulsion T344, respectively. As pH increases above 5, percentage of drug loading decreases. This may be due to fact that the fraction of Betahistine hydrochloride (pKa 9.1) protonation decreases as the pH increases and reduces the interaction with the resin. Complexation was found to be optimum in case of stirring, a maximum of 94.00 ± 0.78 for Tulsion T344, and in case of swelling 93.82 ± 0.87 for Tulsion T344, respectively. This finding may indicate the significant involvement of *van-der-waals* forces taking place along with drug exchange during complexation. Drug resin in the ratio of 1 : 4 gives optimum loading. The drug loading was found to be 92.90 ± 0.59 for Tulsion T344, respectively. Increase in the amount of resin increases the amount of drug adsorbed from the solution. Maximum drug loading on the resin occurs at a temperature of 80°C, a maximum of 94.72 ± 0.69 for Tulsion T344, respectively. Increased temperature during complexation increases ionization of drug and resin. Higher temperatures tend to increase the diffusion rate of ions by decreasing the thickness of exhaustive exchange zone.

The different micromeritic properties resinates like shape, flow properties, bulk density, tap density, and packing ability were studied (Table 2). The results showed that the resinates have good flow properties and packing abilities.

**Figure 1:**
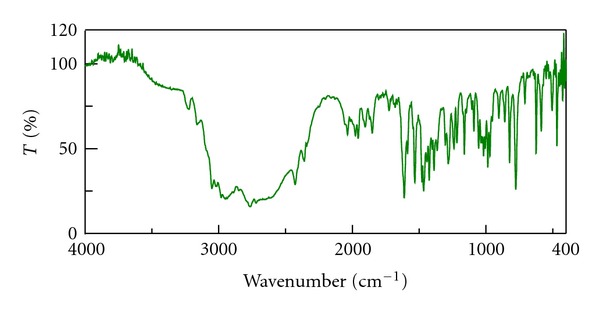
IR spectrum of Betahistine hydrochloride.

**Figure 2:**
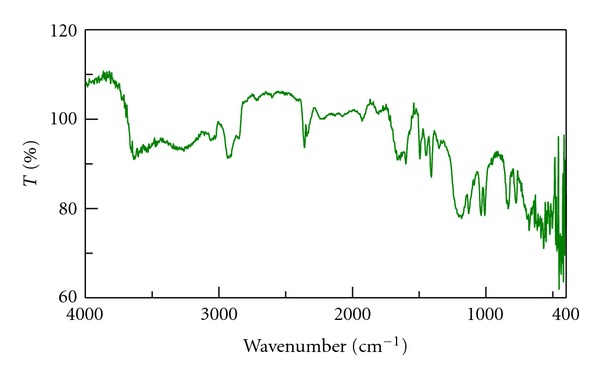
IR spectrum of Tulsion T344.

**Figure 3:**
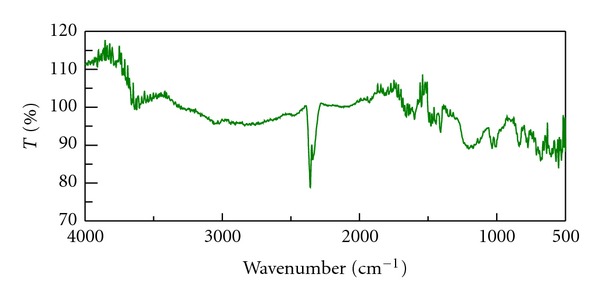
IR spectrum of Tulsion DRC.

The infrared spectra of drug, Tulsion T344 and resinate are shown in Figures 1, 2, and 3. IR spectra of BH show peak at 3212 cm^−1^ which is due to N–H stretching. IR of drug also shows peak at 1349 cm^−1^ due to C=N stretching. The peak at 1600 cm^−1^ is due to C=C stretching. The IR spectra of resinate lack the peak at 1349 cm^−1^ which mainly indicates that the complex between the drug and resin has been formed and tertiary amine group is specifically involved in complex formation.

**Figure 4:**
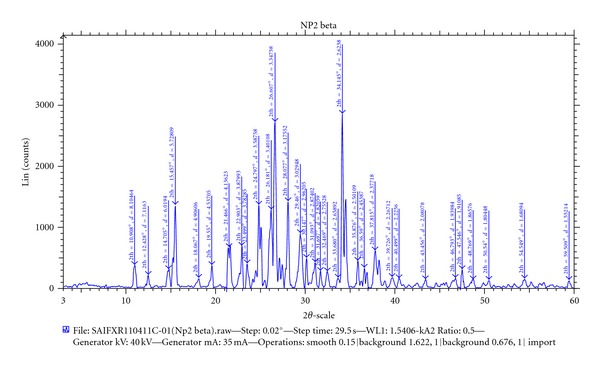
XRPD pattern of Betahistine hydrochloride.

**Figure 5:**
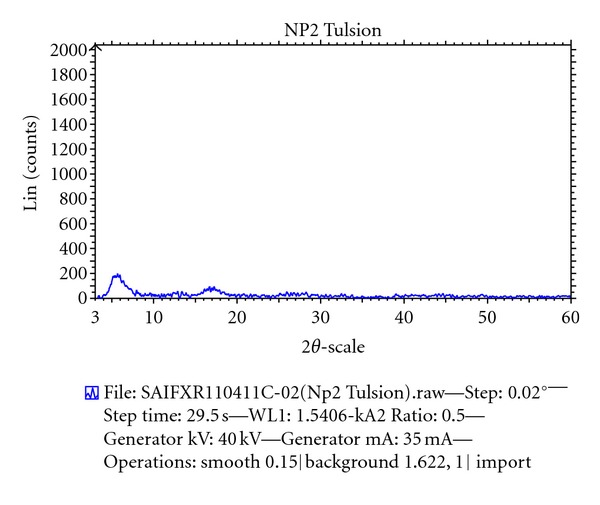
XRPD pattern of Tulsion T344.

**Figure 6:**
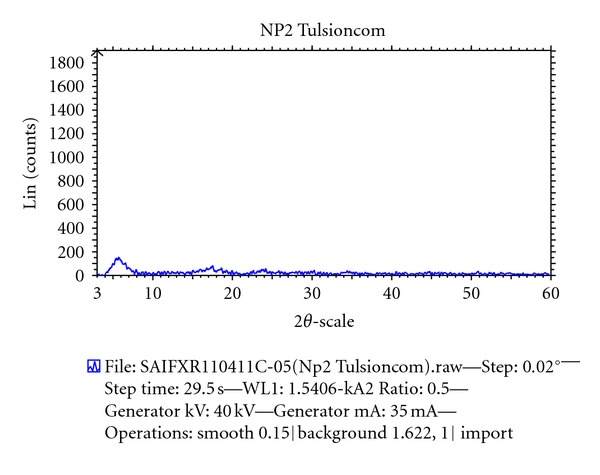
XRPD pattern of Tulsion DRC.

**Figure 7:**
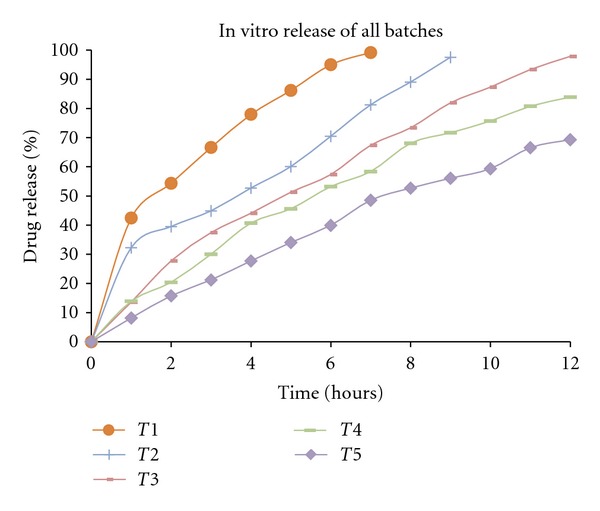
In vitro release of all batches.

The X-ray diffraction pattern for BH contained a number of sharp peaks, while the resin showed a diffused peak, where as only a diffused peak was observed in X-ray powder diffraction patterns for the DRC regardless of drug loading (Figures 4, 5, and 6). According to this data molecular state of BH is crystalline but that of resin is amorphous. The molecular state of BH prepared as drug resin complexes was changed from crystalline state to amorphous state. This shows that entrapped drug molecule is monomolecular dispersed in resin matrix. The prepared batches of tablets were evaluated for various official and nonofficial parameters. Tablets were obtained from uniform weight due to uniform die filled with acceptable variations as per IP (Indian Pharmacopoeia) specifications, that is, below 7.5%. The hardness of tablets for each formulation was between 5-6 kg/cm^2^. Average thickness was found to be in the range of 7 to 8 mm. Friability below 1% was an indication of good mechanical resistance of the tablets. The uniformity of drug content was found to be 97%–99% w/w which was within acceptable limits. Results are shown in Table 3. Results of the in vitro release studies of various formulations designed and manufactured are shown in Figure 7. The result showed that, in case of Tulsion T344 resinate tablet, more than 97% of drug released from tablets formulation with HPMC (K100M) in 30 mg. This may be because of strong binding properties of HPMC which binds the fine particles of resinate. The drug release from these tablets was simply due to slow erosion and ion exchange.

## 4. Conclusion

Tablets formulated with Tulsion T344 and 30 mg HPMC K100M provided sustained release of drug over a period of time, 12 h. The release of Betahistine hydrochloride from resinates controls the diffusion of the drug molecule through the polymeric material into aqueous medium.

## Figures and Tables

**Table 1:** Formulation of tablets.

Ingredients	Batch				
	T1	T2	T3	T4	T5
Tulsion T344 resinate	118^∗^	118^∗^	118^∗^	118^∗^	118^∗^
Microcrystalline cellulose	117	92	87	82	77
HPMC K100M	—	25	30	35	40
PVP K30	7	7	7	7	7
Mg stearate	3	3	3	3	3
Talc	5	5	5	5	5

^∗^DRC containing 24 mg Betahistine and total weight of tablet is 250 mg.

**Table 2:** Physical properties of Tulsion 344 and DRC.

Character	Tulsion 343	DRC
Shape	Irregular	Irregular
Angle of repose (^°^)	27.98 ± 0.97	30.2 ± 0.45
Bulk density	0.635 ± 0.005196	0.675 ± 0.04
Tapped density	0.744 ± 0.03	0.785 ± 0.06
Carr's index	14.64 ± 0.606987	13.96 ± 0.98
Hausner ratio	1.156 ± 0.23	1.16 ± 0.17

**Table 3:** Evaluation parameter of tablets.

Parameters batches	Weight variation (mg) (*n* = 3)	Hardness (kg/cm^2^) (*n* = 3)	Friability (% w/w)	Thickness (mm) (*n* = 3)	Drug content (*n* = 3)
T1	247 ± 1.90	5.8 ± 0.05	0.65 ± 0.37	7.2 ± 0.34	99.69 ± 0.64
T2	249 ± 1.12	5.7 ± 0.04	0.59 ± 0.46	7.0 ± 1.08	98.48 ± 0.68
T3	248 ± 0.43	5.2 ± 0.06	0.48 ± 0.27	7.3 ± 0.94	97.79 ± 0.75
T4	247 ± 0.86	5.4 ± 0.07	0.57 ± 0.52	7.2 ± 0.79	95.96 ± 0.35
T5	251 ± 0.19	5.6 ± 0.13	0.68 ± 0.27	7.1 ± 0.33	98.72 ± 0.84
